# Changes in microbial ecology after fecal microbiota transplantation for recurrent *C. difficile* infection affected by underlying inflammatory bowel disease

**DOI:** 10.1186/s40168-017-0269-3

**Published:** 2017-05-15

**Authors:** Sahil Khanna, Yoshiki Vazquez-Baeza, Antonio González, Sophie Weiss, Bradley Schmidt, David A. Muñiz-Pedrogo, John F. Rainey, Patricia Kammer, Heidi Nelson, Michael Sadowsky, Alexander Khoruts, Stefan L. Farrugia, Rob Knight, Darrell S. Pardi, Purna C. Kashyap

**Affiliations:** 1 Division of Gastroenterology and Hepatology, Mayo Clinic, Rochester, MN 55905 USA; 2 Department of Computer Science and Engineering, University of California, San Diego, La Jolla, CA USA; 3 Department of Pediatrics, University of California, San Diego, La Jolla, CA USA; 4 Department of Chemical and Biological Engineering, University of Colorado, Boulder, CO USA; 5 Division of Colorectal Surgery, Mayo Clinic, Rochester, MN USA; 6 BioTechnology Institute, University of Minnesota, Minneapolis, MN USA; 7 Division of Gastroenterology, University of Minnesota, Minneapolis, MN USA

**Keywords:** Fecal microbiota transplantation, Microbiome, *Clostridium difficile* infection, Inflammatory bowel disease

## Abstract

**Background:**

Gut microbiota play a key role in maintaining homeostasis in the human gut. Alterations in the gut microbial ecosystem predispose to *Clostridium difficile* infection (CDI) and gut inflammatory disorders such as inflammatory bowel disease (IBD). Fecal microbiota transplantation (FMT) from a healthy donor can restore gut microbial diversity and pathogen colonization resistance; consequently, it is now being investigated for its ability to improve inflammatory gut conditions such as IBD. In this study, we investigated changes in gut microbiota following FMT in 38 patients with CDI with or without underlying IBD.

**Results:**

There was a significant change in gut microbial composition towards the donor microbiota and an overall increase in microbial diversity consistent with previous studies after FMT. FMT was successful in treating CDI using a diverse set of donors, and varying degrees of donor stool engraftment suggesting that donor type and degree of engraftment are not drivers of a successful FMT treatment of CDI. However, patients with underlying IBD experienced an increased number of CDI relapses (during a 24-month follow-up) and a decreased growth of new taxa, as compared to the subjects without IBD. Moreover, the need for IBD therapy did not change following FMT. These results underscore the importance of the existing gut microbial landscape as a decisive factor to successfully treat CDI and potentially for improvement of the underlying pathophysiology in IBD.

**Conclusions:**

FMT leads to a significant change in microbial diversity in patients with recurrent CDI and complete resolution of symptoms. Stool donor type (related or unrelated) and degree of engraftment are not the key for successful treatment of CDI by FMT. However, CDI patients with IBD have higher proportion of the original community after FMT and lack of improvement of their IBD symptoms and increased episodes of CDI on long-term follow-up.

**Electronic supplementary material:**

The online version of this article (doi:10.1186/s40168-017-0269-3) contains supplementary material, which is available to authorized users.

## Background

Gut microbiota play a key role in maintaining homeostatic host functions, and deleterious shifts in the gut microbial ecosystem, often referred to as dysbiosis, are associated with *Clostridium difficile* infection (CDI), inflammatory bowel disease (IBD), and other systemic inflammatory conditions [1]. A diverse gut microbial community confers colonization resistance against pathogens such as *C. difficile*, and disruption of a diverse community structure from antibiotics, comorbidities, altered gastrointestinal transit, or other risk factors can lead to pathogen colonization and infection [2].

With increasing incidence of community and hospital acquired CDI, high rates of recurrent CDI (estimated 20–30% after a first and 50–60% after a third infection), high mortality (~29,000 deaths annually) in the USA, and an urgent need for newer non-antibiotic therapies has led to the emergence of microbiome-based therapies [3]. Fecal microbiota transplantation (FMT) in CDI patients restores phylogenetic diversity to levels more typical of a healthy person, with response rates >85% by enema, oral capsule, or endoscopic delivery modes [4–6]. A recent study suggests significantly lower response of CDI to FMT in patients with underlying inflammatory bowel disease (IBD) [7]. We have also previously described a higher rate of recurrence of CDI following FMT in patients with CDI and underlying IBD [8]. It remains unclear if changes in gut microbial ecology play a role in long-term success of FMT in these patients.

FMT has not shown consistent success in treating other diseases associated with microbial dysbiosis such as IBD. Three clinical trials to treat ulcerative colitis (UC) with FMT have shown conflicting results, and one highlighted the potential role of specific gut microbial members in donor stool in determining success after FMT in UC [9–11]. The underlying host or donor factors that may be important for success of FMT in treatment of IBD remain unclear.

In this study, we assessed the effect of donor type (standard donor versus related donor) and changes in gut microbial ecology on response to FMT in recurrent CDI with and without underlying IBD as well as clinical response to FMT.

## Methods

### Patient selection

Patients undergoing FMT for recurrent CDI were prospectively recruited in this study. Informed consent was obtained to collect clinical data and stool samples. Data collected included demographics, clinical history, CDI treatment history, comorbid conditions, and response to FMT. A donor fecal sample was collected prior to FMT. Stool samples from the recipients were collected before FMT, and at day 7 and day 28, and were stored at −80 °C. The donors were either related (genetically related family members) or unrelated (screened hospital employee volunteer donors or unrelated family members), and a fresh sample was obtained on the day of FMT. All donors underwent extensive screening in accordance with standard practice and guidelines from the Food and Drug Administration [12]. Donor selection criteria and experience from our group have been previously published [13]. The donor stool sample is weighed and divided into 50 g aliquots. Each aliquot of 50 g is diluted in normal saline in a 1:5 ratio (50 g of stool diluted with 250 ml of normal saline) and is placed in the blender bag (a two-bag system with a semipermeable membrane in the inner bag and the outside bag is plastic). The stool is placed in the inner bag and normal saline is added. The bag is placed in a sealed compartment in the stomacher 400 (Seward) and blended for 60 s at 230 rotations per minute. The filtrate is then placed into 50 ml conical tubes using 50 ml pipettes and placed on an ice pack prior to the procedure. Recurrent CDI was defined as another episode of CDI within 56 days after symptom resolution with recurrence of symptoms and a positive stool polymerase chain reaction test. For this study, future *C. difficile* episodes after FMT up to 2 years were captured. These were categorized as up to 56 days, 56 days to 1 year, and beyond 1 year.

### Sequencing and analytic methods

After fecal DNA isolation (MoBio, Carlsbad, CA fecal DNA kit), amplicons spanning the variable region 4 of bacterial 16S rRNA were generated and sequenced using Illumina MiSeq platform at the Mayo Clinic Medical Genome Facility, Rochester, MN. The 16S rRNA sequencing data from the Illumina runs were quality controlled, trimmed, demultiplexed, and assigned to operational taxonomic units (OTUs) following the closed reference at 97% similarity (using SortMeRNA as a clustering algorithm [14] protocol against the Greengenes [15] database 13_8 release, as implemented in Quantitative Insights Into Microbial Ecology (QIIME) 1.9.0 software [16], default parameters were used for all these steps unless otherwise noted. After quality control, 10,583,052 sequences were obtained, for a mean of 76,688 sequences per sample (min: 33,559, max: 154,200).

Alpha diversity values were calculated using Faith’s phylogenetic diversity [17]. To assess differential abundance between the groups, we used ANCOM [18], as implemented in scikit-bio 0.5.1 (http://scikit-bio.org/docs/0.5.1/). This is tested by looking at the individual OTUs across the patient types (with and without underlying IBD); OTUs of the same genus are grouped for displaying purposes. We note that ANCOM makes the statistical assumption that fewer than 25% of taxa change, not met in all these comparisons (before FMT and post FMT communities are expected to be very different [19]). The donor plane is created using all the donor samples and serves as a proxy for where their microbiomes are in the ordination space, and how as time goes by this proximity changes. This procedure was originally presented by Halfvarson et al. [20].

Beta diversity matrices were created using unweighted UniFrac [21] and plotted using Emperor [22] (all other plotting was done using the Seaborn visualization package).

Processed tables and sample information can be found in Qiita (https://qiita.ucsd.edu) under study id 10057; alternatively, the data can be found under accession number ERP021216 at the European Bioinformatics Institute.

### SourceTracker analysis

To assess the proportion of pre-transplant communities that were retained in the patients’ microbiota, we used SourceTracker [23]. The pre-transplant samples and the donor samples were described as *sources*; all the other samples were used as *sinks*. For all samples at days 7 and 28, SourceTracker estimated the proportion of communities that were attributed 1 of 3 environments, (1) the donor, (2) the patient pre-transplant, and (3) unknown community. Using these proportions, we grouped the samples according to their IBD status and compared their distributions using the Mann-Whitney test (as implemented in SciPy 0.15.1 [24]).

### Clinical statistics

Statistical analyses for clinical data were performed with JMP version 11.0 (SAS institute, NC). Data analysis included descriptive statistics, *t* tests for normally distributed variables, non-parametric tests for skewed variables, chi-square tests, and ANOVA tests as applicable. A *p* value less than 0.05 was considered statistically significant.

## Results

### FMT leads to resolution of CDI

In order to assess gut microbiota changes following FMT, 38 patients with recurrent CDI were enrolled in the study and a fecal sample was obtained prior to transplant, as well as 7 and 28 days post-transplant. Sample handling, donor and recipient sample collection, sample processing, and data analyses are detailed in supplementary methods. FMT was accomplished by colonoscopy using fresh donor stools from related (*n* = 12) or unrelated (*n* = 26) donors. None of the IBD patients received stool from a related donor. The demographic, disease, and treatment characteristics are outlined in Table 1. Detailed characteristics of IBD patients are shown in Additional file 1: Table S1. Twelve patients (31.6%) had IBD (6 with UC and 6 with Crohn’s disease), with median age 27.6 years (range 23.3–74.9), and median IBD duration 5 years (range 2–33). 58.3% of patients were on 5-ASA (amino salicylic acid) agents, 50% on biologics, 33.3% on immunomodulators, and 58.3% on steroids. Among patients with IBD, at the time of colonoscopy, 2 had normal colonoscopy, 1 had pseudopolyps, 5 had severe pancolitis, 1 had moderate colitis, 1 had mild colitis, 1 had mild procto-sigmoiditis, and 1 had moderate ileo-colitis (Additional file 1: Table S1).

**Table 1:** Clinical characteristics

	Overall (*n* = 38)	IBD (*n* = 12)	No IBD (*n* = 26)
Age, median (range)	53.1 (21.9–82.7)	27.6 (23.3–74.9)	58.3 (21.9–82.7)
Sex distribution (% female)	81.6	66.7	88.5
BMI, kg/m^2^ median (range)	24.8 (14.9–39.9)	25.6 (18.5–30.3)	23.8 (14.9–39.9)
Number prior CDI episodes, median (range)	5 (3–13)	4.5 (3–7)	5 (3–13)
Number prior metronidazole courses, median (range)	1 (0–8)	1 (0–2)	1 (0–8)
Patients treated with at least one prior course of metronidazole, *n* (%)	33 (86.8%)	9 (75%)	24 (92.3%)
Number prior vancomycin 10–14 day courses, median (range)	2 (0–4)	2 (0–4)	2 (0–3)
Patients treated with at least one prior course of vancomycin, *n* (%)	36 (94.7%)	11 (91.7%)	25 (96.2%)
Number prior vancomycin tapers, median (range)	1 (0–5)	1 (0–1)	1 (0–5)
Patients treated with at least one prior course of vancomycin taper, *n* (%)	26 (68.4%)	7 (58.3%)	19 (73.1%)
Number prior fidaxomicin courses, median (range)	0 (0–4)	0 (0–2)	0 (0–4)
Patients treated with at least one prior course of fidaxomicin, *n* (%)	16 (61.5%)	5 (41.7%)	16 (61.5%)
Related donors, *n* (%)	12 (31.6%)	0 (0%)	12 (46.2%)
Recurrent CDI within 24 months of FMT, *n* (%)	5 (13.2%)	3 (25%)	2 (7.7%)

All patients responded to FMT with regards to clinical or microbiologic remission of CDI (negative *C. difficile* testing in the presence of ongoing diarrhea), 92.1% (*n* = 35) of patient symptoms returned to baseline bowel pattern (as before CDI) and resolution of CDI, 5.3% (*n* = 2, both with IBD) had worsening diarrhea (*C. difficile* negative), and 2.6% (*n* = 1) had new onset constipation after FMT. Upon long-term follow-up of 24 months; 13.2% (*n* = 5/38; of these, *n* = 1 within 56 days, *n* = 1 from 56 days to 1 year, and *n* = 3 beyond 1 year, Additional file 1: Table S2) had another episode of CDI and 10.5% (*n* = 4/38) required a second FMT due to multiply recurrent CDI. One patient with rCDI was treated with vancomycin. The risk of another episode of CDI after FMT in IBD patients was 25% (*n* = 3/12) compared to 7.7% (*n* = 2/26) in non-IBD patients (*p* = 0.16, chi-square test). Seven of the 12 patients with IBD were on systemic immunosuppression. None of the patients with IBD had improvement in their IBD course after FMT, and none were able to withhold, de-escalate, or stop IBD treatment. This is in agreement with other studies showing a lack of improvement in IBD following a single FMT.

### FMT decreases microbial dysbiosis

**Fig. 1:**
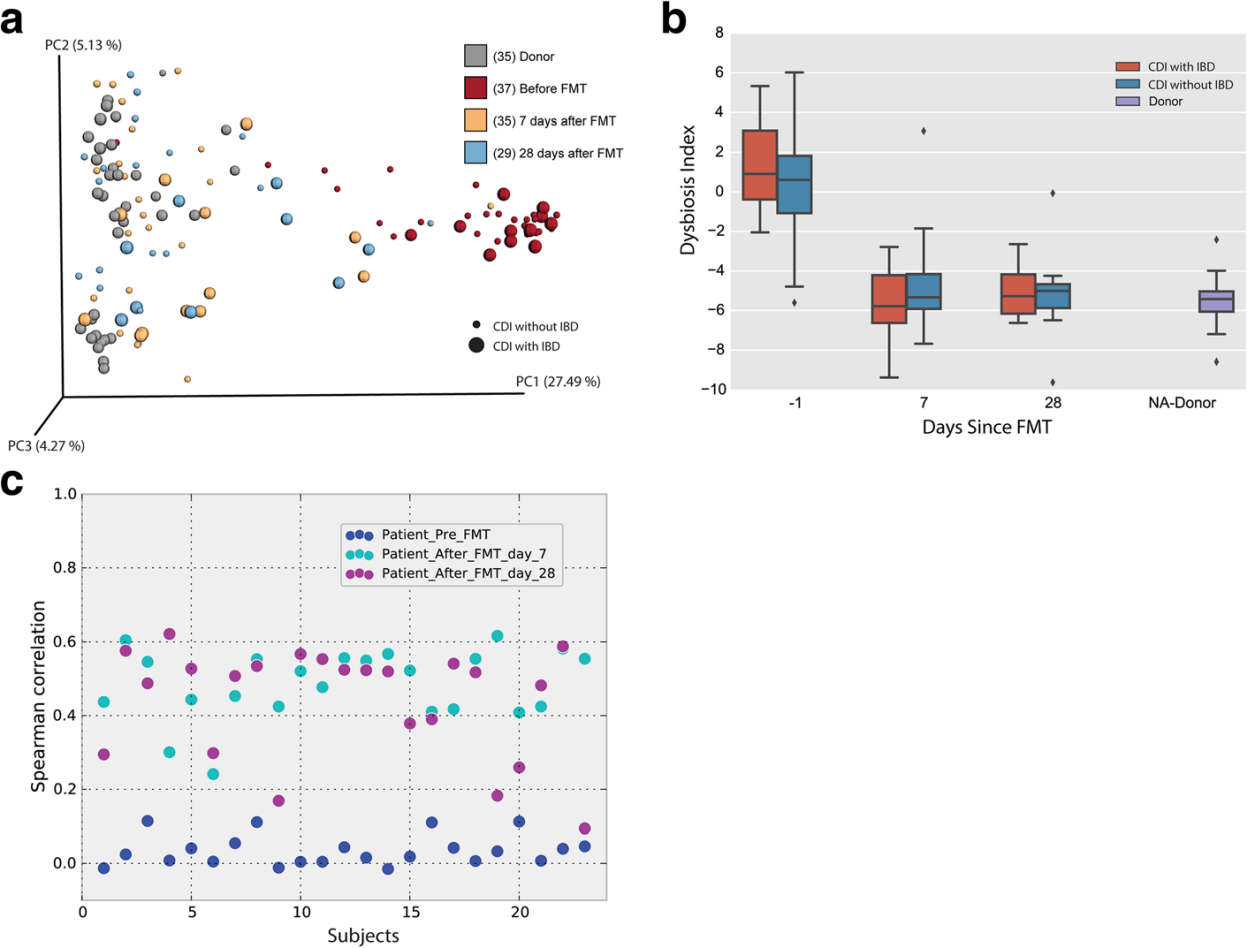
**a** Principal Coordinates Analysis of the unweighted UniFrac distances, showing a significant change in the phylogenetic diversity between patients with CDI, 7 and 28 days after fecal microbiota transplant (PERMANOVA *p* < 0.05). **b** Change in dysbiosis index following fecal microbiota transplant in patients with CDI with or without IBD, demonstrating that the microbial dysbiosis index values were significantly higher in patients with CDI compared to donors (Mann-Whittney’s *U*, *p* < 0.05). **c** Spearman correlation to donor stool 7 and 28 days following fecal microbiota transplantation demonstrating that the fecal microbial communities from patients with CDI were distinct from donor communities prior to transplant (Spearman’s *r* < 0.2 for all subjects)

FMT led to a significant increase in alpha diversity based on Faith’s phylogenetic diversity, Shannon’s diversity index, and observed species, both at day 7 and day 28 (Mann-Whitney *p* < 0.05; Additional file 2: Figure S1, comparing pre- and post-FMT in patients with CDI with or without underlying IBD). Also, patient’s stool closely resembled donor stool, as evidenced by a rapid and sustained change in unweighted and weighted UniFrac-based beta diversity following FMT at day 7 and 28 post-transplant (Fig. 1a; PERMANOVA *p* < 0.05) [21].

To characterize the changes in community composition, we use the microbial dysbiosis index (MD index) as a reference to describe the dominance of individual taxa (Additional file 1: Table S3). The MD index is composed of 18 taxonomic groups, as defined by Gevers et al., with a higher value correlated with greater disease severity in IBD and lower values associated with healthier states [25]. As CDI is also associated with dysbiosis and inflammation, we wanted to determine the effect of FMT on dysbiosis. The MD index values were significantly higher in patients with CDI compared to donors (Mann-Whittney’s U, *p* < 0.05, Fig. 1b). However, on days 7 and 28 after the transplantation, the MD index values were similar to donors (Mann-Whitney’s U *p* > 0.05, Fig. 1b) and this change was independent of whether recipients had IBD or not.

In order to determine if the changes seen in our subjects following FMT were similar to other published studies, we compared our samples with recently published data from Weingarden et al. 2015 (Additional file 3: Figure S2A) wherein four patients with recurrent CDI (but not IBD) received FMT from a single donor [19]. Similar to our findings, there was a rapid and sustained change in beta diversity (Additional file 3: Figure S2A) following FMT and the regression to the donor plane (change in microbial composition to resemble healthy donors) following FMT was remarkably similar in the two studies (Additional file 3: Figure S2B). In this context, we refer to the donor plane as a proxy to the region in the Principal Coordinates Analysis (PCoA; a dimensionality reduction method to visualize beta-diversity distance matrices) space where the donors are located; we do this by fitting a three-dimensional plane (using the least squares method) to the samples from the donors. As the communities change post-FMT, the distance to this plane is reduced.

### Clinical response of CDI to FMT is independent of engraftment or donor type but underlying IBD influences changes in gut microbial ecology after FMT

**Fig. 2:**
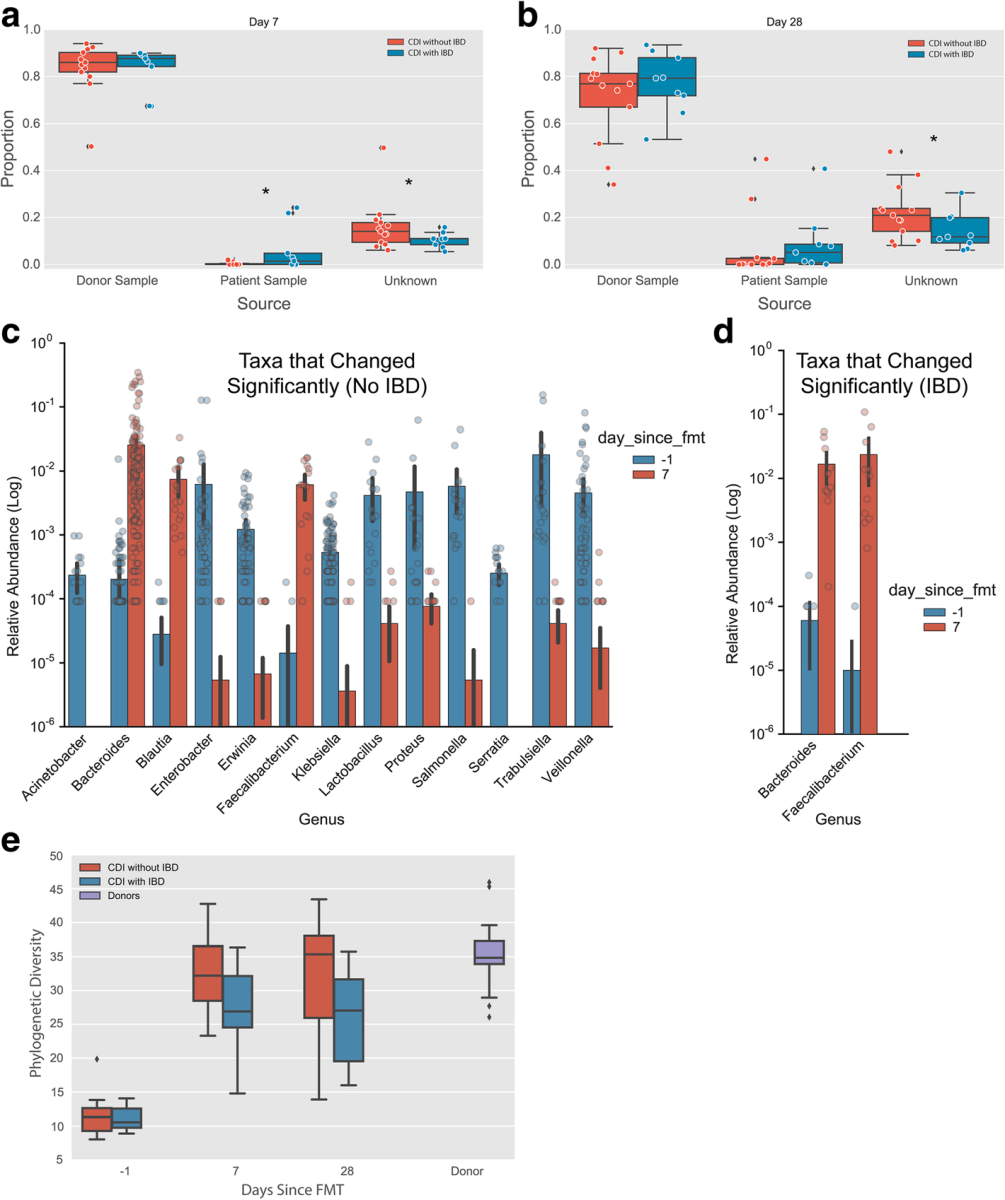
**a**, **b** Subjects with IBD retain a higher proportion of their original communities (*Mann-Whitney *p* < 0.05 at day 7 and *p* = 0.06 at day 28, and a significantly lower proportion of new communities (*Mann-Whitney *p* < 0.05 at days 7 and 28), as compared to the patients without IBD using SourceTracker. **c** Bacterial taxa that change significantly in patients with IBD after FMT (ANCOM *p* < 0.05, corrected for multiple comparisons using Bonferroni-Holm’s method). **d** Bacterial taxa that change significantly in patients without IBD after FMT (ANCOM *p* < 0.05, corrected for multiple comparisons using Bonferroni-Holm’s method). **e** Change in phylogenetic diversity-based alpha diversity 7 and 28 days following fecal microbiota transplant in patients with CDI with and without IBD (Mann-Whitney’s *U p* < 0.001)

In order to determine if the response of CDI to FMT was dependent on donor stool engraftment, we determined Spearman’s correlation coefficient between fecal microbial communities prior to 7 and 28 days post-transplant. The fecal microbial communities from patients with CDI were distinct from donor communities prior to transplant (Spearman’s *r* < 0.2 for all subjects, Fig. 1c). Following transplant, communities showed an increase in correlation to donor stool at day 7 (Spearman’s *r* > 0.4 for 85% of the subjects, Fig. 1c) and a spread for all subjects at day 28 ranging from below 0.2 up to 0.6 (Fig. 1c). Using SourceTracker [23], we found that after FMT, subjects with IBD retained a higher proportion of their original communities (Mann-Whitney *p* < 0.05 at day 7, and *p* = 0.06 at day 28; Fig. 2a, b) and a significantly lower proportion of new communities (Mann-Whitney *p* < 0.05 at days 7 and 28), as compared to the patients without IBD. The expansion of new taxa following FMT as seen in patients without IBD may represent a beneficial ecological change following FMT; however, future studies will be needed to address the biological effect of newly acquired community members following FMT. Consequently, in patients with IBD, we observed a smaller group of taxa that change significantly 7 days after FMT. In both groups, *Bacteroides* and *Faecalibacterium* showed a significant increase in relative abundance, with *Blautia*, only being increased for patients without IBD. Additionally, these patients showed a decrease in relative abundance of *Lactobacillus*, *Veillonella*, *Enterobacter*, *Klebsiella*, *Erwina, Proteus*, *Salmonella*, and *Trabulsiella* (Fig. 2c, d, ANCOM *p* < 0.05, corrected for multiple comparisons using Bonferroni-Holm’s method [18]).

All patients had either clinical or microbiological remission (negative *C. difficile* testing in the presence of ongoing diarrhea) confirming that initial response of CDI to FMT is not dependent on the degree of donor stool engraftment. In this small cohort of patients, those with underlying IBD had higher number of late relapses of CDI. We found no significant differences in gut microbiota composition following FMT from standard donors or related donors (Mann-Whitney *p* > 0.05 at days 7 and 28), suggesting that engraftment of donor stool was independent of donor type. Furthermore, as all patients had ongoing clinical remission with microbiological response (if measured), donor type does not appear to affect CDI-related clinical response.

### Change in bacterial diversity after FMT is dependent on underlying IBD

IBD disease course, as measured by the need for specific IBD therapies, did not change after FMT, and patients with CDI and underlying IBD retained a higher proportion of the pre-transplant communities and lower proportion of new communities following FMT. Thus, underlying IBD appears to affect the change in gut microbial ecology resulting in a less significant increase in overall diversity. In subjects without IBD, Faith’s phylogenetic diversity (which measures the total branch length of a phylogenetic tree that a given sample covers [26]) reached a level comparable to healthy donors (Mann-Whitney’s *U p* < 0.001, Fig. 2e). The differences in phylogenetic diversity following FMT between subjects with and without IBD became evident on day 7 and persisted on day 28 (Mann-Whitney, day 1 *p* = 0.163, day 7 *p* = 0.0058, and day 27 *p* = 0.008, Fig. 2e). A linear regression of phylogenetic diversity versus MD index (Additional file 4: Figure S3) shows a significantly lower negative correlation between the increase in phylogenetic diversity and the increase of the MD index in patients with IBD (Pearson’s correlation coefficient, IBD *R* = −0.68, no IBD *R* = −0.83; *p* < 0.0001; Additional file 4: Figure S3) suggesting a lack of recovery of phylogenetic diversity in patients with IBD as the MD index improves.

## Discussion

In this study, we found that gut microbiota diversity changes rapidly following FMT for treatment of CDI and resembles donor microbiota diversity, similar to previous studies. A successful response of CDI to FMT was seen with a diverse group of donors and at levels of engraftment (as measured by correlation to donor stool) varying from 50 to 94% (at day 7) and 34–93% (at day 28) based on the proportion of communities attributed to the donor following FMT per SourceTracker, suggesting these are not critical factors in determining response. Similarly, a recent study that evaluated pre- and post-FMT (for recurrent CDI) gut microbiome samples from a subset of patients enrolled in a randomized controlled trial [27], compared donor FMT to autologous FMT suggested that complete engraftment of donor bacteria may be not necessary, if functionally, critical taxa are present in subjects following initial antibiotic therapy for CDI [28]. This study excluded patients with IBD but was able to compare autologous to donor FMT unlike our study. There was a higher number of recurrent CDI following FMT in patients with CDI and IBD, but this was not statistically significant, likely given the small sample size. However, we have previously reported similar findings in a larger cohort of patients with CDI and IBD [8], where gut microbiota changes were not monitored. Interestingly, in this cohort, all patients had an initial clinical or microbiological remission (negative *C. difficile* testing in the presence of ongoing diarrhea) of CDI following FMT, and we did not see a difference in initial response reported in a recent study [7], which is also likely due to the smaller sample size of our study and differences in underlying disease characteristics.

We also did not see changes in need for IBD therapy in the subset of patients with IBD underlying CDI. While dynamic variations can be seen in patients following FMT [19], patients with underlying IBD in our study show a higher proportion of the original pre-transplant microbial community and lower recovery of phylogenetic diversity following FMT compared to those without IBD. This lack of beneficial change in microbial ecology may be relevant for long-term response of CDI in patients with IBD and the lack of clinical response of IBD to FMT seen in our and previous studies [7]. Future studies designed to study the effect of compositional and functional changes in gut microbiota on clinical outcomes following FMT in patients with IBD will be needed to definitively address the potential importance of changes in microbial ecology, donor selection [9], underlying disease characteristics, and multiple-dose FMTs, in correcting the underlying pathophysiology of IBD.

## Conclusions

There is a significant increase in microbial diversity in patients with recurrent CDI after FMT. Both the degree of microbial engraftment or donor type (related or unrelated) are not the key for successful treatment of recurrent CDI by FMT. Compared to CDI patients without IBD, CDI patients with IBD have higher proportion of the original microbial communities after FMT and increased episodes of future CDI on long-term follow-up.

## Additional files

Additional file 1: Table S1. Clinical characteristics of patients with inflammatory bowel disease. **Table S2:** *Clostridium difficile* infection episodes after fecal microbiota transplantation. **Table S3:** Bacterial taxa comprising the microbial dysbiosis index. (DOCX 64 kb)

Additional file 2: Figure S1. A significant increase in alpha diversity in patients with CDI following FMT using phylogenetic diversity, Shannon diversity, and observed species (Mann-Whitney *p* < 0.05). (TIF 221 kb)

Additional file 3: Figure S2. (A) Meta-analysis showing changes in unweighted UniFrac-based beta diversity in a published cohort in comparison with our cohort. (B) Phylogenetic diversity regressions against the healthy plane (where the healthy plane is defined as a surface that’s fitted to the first 3 dimensions of the coordinates of the healthy samples). (TIF 1127 kb)

Additional file 4: Figure S3. Linear regression of dysbiosis index versus phylogenetic diversity in patients with CDI with and without IBD demonstrating a significantly lower negative correlation between the increase in phylogenetic diversity and the increase of the microbial dysbiosis index in patients with IBD (Pearson’s correlation coefficient, IBD *R* = −0.68, no IBD *R* = −0.83; *p* < 0.0001). (TIF 769 kb)

## Abbreviations

CDI: *Clostridium difficile* infection
FMT: Fecal microbiota transplantation
IBD: Inflammatory bowel disease
MD: Microbial dysbiosis
OTU: Operational taxonomic units
PCoA: Principal Coordinates Analysis
QIIME: Quantitative Insights Into Microbial Ecology
UC: Ulcerative colitis
