# Supplementary material for: Changes in microbial ecology after fecal microbiota transplantation for recurrent C. difficile infection affected by underlying inflammatory bowel disease
Source: Microbiome. 2017 May 15;5:55. doi: 10.1186/s40168-017-0269-3 (PMC5433077; doi:10.1186/s40168-017-0269-3)
Supplement: Supplementary file 1 — Clinical characteristics of patients with inflammatory bowel disease. Table S2: Clostridium difficile infection episodes after fecal microbiota transplantation. Table S3: Bacterial taxa comprising the microbial dysbiosis index. (DOCX 64 kb) [file 40168_2017_269_MOESM1_ESM.docx]

**Supplementary Table 1:** Clinical characteristics of patients with Inflammatory bowel disease

|  | Crohn’s disease  (n=6) | Ulcerative colitis  (n=6) |
| --- | --- | --- |
| Disease extent  Ileum  Pan-colitis  Procto-sigmoiditis | 0  6  0 | N/A  4  2 |
| Disease state  Remission (Normal)  Mayo score 1  Mayo score 2  Mayo score 3 | 2  0  2  2 | 1  1  1  3 |
| 5-Aminosalicylic acid agents | 2 | 3 |
| Steroids | 3 | 3 |
| Immunomodulators | 3 | 1 |
| Biologics | 3 | 2 |

**Supplementary Table 2:** *Clostridium difficile* Infection Episodes after Fecal Microbiota Transplantation

|  | Within 56 days | 56 days to 1 year | > 1year |
| --- | --- | --- | --- |
| All Patients  (n = 38) | 1 | 1 | 3 |
| CDI with IBD  (n = 12) | 0 | 1 | 2 |
| CDI without IBD  (n =26) | 1 | 0 | 1 |

**Supplementary Table 3:** Bacterial taxa comprising the Microbial dysbiosis index

| Veillonella | Increased in CD |
| --- | --- |
| Escherichia | Increased in CD |
| Haemophilus | Increased in CD |
| Fusobacterium | Increased in CD |
| Bialister | Decreased in CD |
| Sutterella | Decreased in CD |
| Rikenellaceae | Decreased in CD |
| Parabacteroides | Decreased in CD |
| Bacteroides | Decreased in CD |
| Lachnospiraceae | Decreased in CD |
| Coprococcus | Decreased in CD |
| Ruminococcus | Decreased in CD |
| Erysipelotrichaceae | Decreased in CD |
| Dorea | Decreased in CD |
| Ruminococcaceae | Decreased in CD |
| Faecalibacterium | Decreased in CD |
| Oscillospira | Decreased in CD |
| Bilophila | Decreased in CD |

CD = Crohn’s disease
