# Supplementary figures and images for: Changes in microbial ecology after fecal microbiota transplantation for recurrent C. difficile infection affected by underlying inflammatory bowel disease
Source: Microbiome. 2017 May 15;5:55. doi: 10.1186/s40168-017-0269-3 (PMC5433077; doi:10.1186/s40168-017-0269-3)

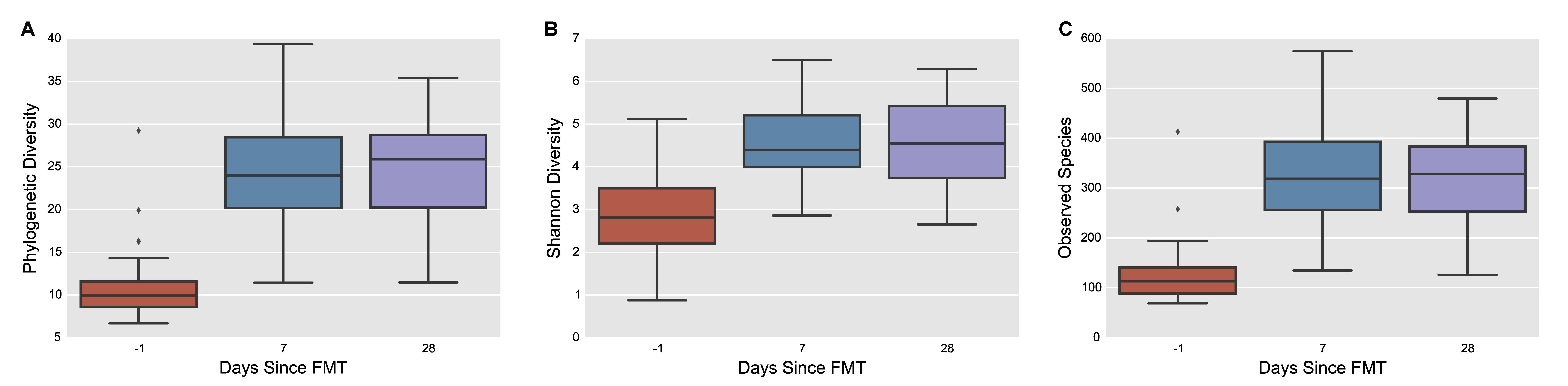

Supplement: Supplementary file 2 — A significant increase in alpha diversity in patients with CDI following FMT using phylogenetic diversity, Shannon diversity, and observed species (Mann-Whitney p < 0.05). (TIF 221 kb) [file 40168_2017_269_MOESM2_ESM.tif]

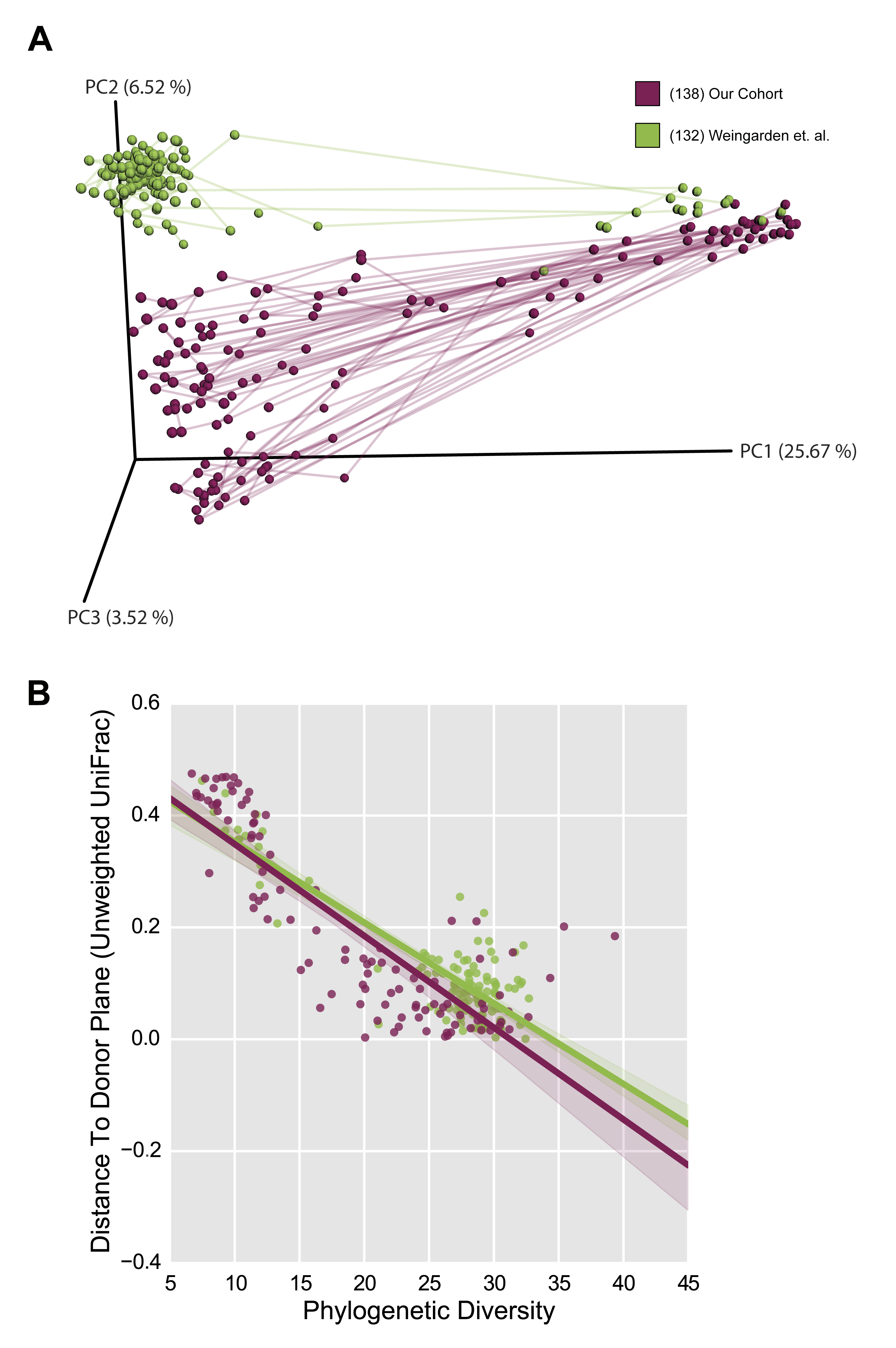

Supplement: Supplementary file 3 — (A) Meta-analysis showing changes in unweighted UniFrac-based beta diversity in a published cohort in comparison with our cohort. (B) Phylogenetic diversity regressions against the healthy plane (where the healthy plane is defined as a surface that’s fitted to the first 3 dimensions of the coordinates of the healthy samples). (TIF 1127 kb) [file 40168_2017_269_MOESM3_ESM.tif]

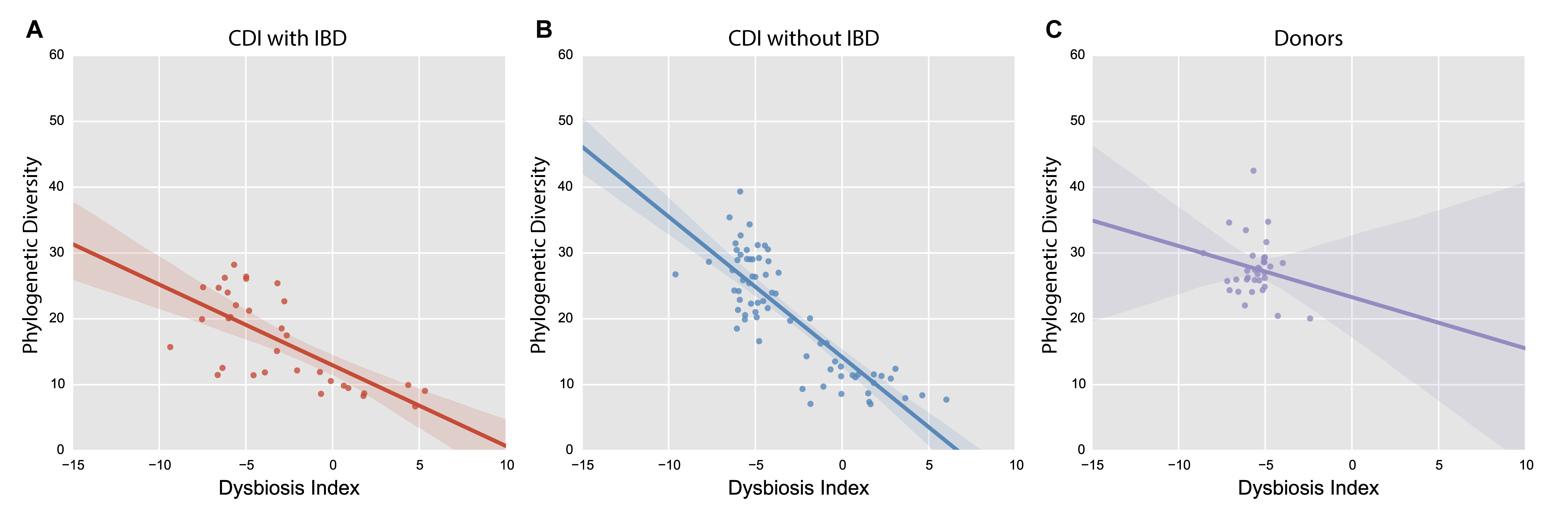

Supplement: Supplementary file 4 — Linear regression of dysbiosis index versus phylogenetic diversity in patients with CDI with and without IBD demonstrating a significantly lower negative correlation between the increase in phylogenetic diversity and the increase of the microbial dysbiosis index in patients with IBD (Pearson’s correlation coefficient, IBD R = −0.68, no IBD R = −0.83; p < 0.0001). (TIF 769 kb) [file 40168_2017_269_MOESM4_ESM.tif]
